# Synthesis and Characterization of Rice Straw/Fe_3_O_4_ Nanocomposites by a Quick Precipitation Method

**DOI:** 10.3390/molecules18066597

**Published:** 2013-06-05

**Authors:** Roshanak Khandanlou, Mansor Bin Ahmad, Kamyar Shameli, Katayoon Kalantari

**Affiliations:** 1 Department of Chemistry, Faculty of Science, Universiti Putra Malaysia, Serdang UPM 43400, Selangor, Malaysia; E-Mails: roshanak_bch@yahoo.com (R.K.); ka_kalantary@yahoo.com (K.K.); 2 Nanotechnology and Advance Materials Department, Materials & Energy Research Center, Karaj, Alborz, 31787/316, Iran

**Keywords:** rice straw, nanocomposites, iron oxide, X-ray powder diffraction, transmission electron microscopy

## Abstract

Small sized magnetite iron oxide nanoparticles (Fe_3_O_4_-NPs) with were successfully synthesized on the surface of rice straw using the quick precipitation method in the absence of any heat treatment. Ferric chloride (FeCl_3_·6H_2_O), ferrous chloride (FeCl_2_·4H_2_O), sodium hydroxide (NaOH) and urea (CH_4_N_2_O) were used as Fe_3_O_4_-NPs precursors, reducing agent and stabilizer, respectively. The rice straw fibers were dispersed in deionized water, and then urea was added to the suspension, after that ferric and ferrous chloride were added to this mixture and stirred. After the absorption of iron ions on the surface layer of the fibers, the ions were reduced with NaOH by a quick precipitation method. The reaction was carried out under N_2_ gas. The mean diameter and standard deviation of metal oxide NPs synthesized in rice straw/Fe_3_O_4_ nanocomposites (NCs) were 9.93 ± 2.42 nm. The prepared rice straw/Fe_3_O_4_-NCS were characterized using powder X-ray diffraction (PXRD), transmission electron microscopy (TEM), scanning electron microscopy (SEM), energy dispersive X-ray fluorescence (EDXF) and Fourier transforms infrared spectroscopy (FT‒IR). The rice straw/Fe_3_O_4_-NCs prepared by this method have magnetic properties.

## 1. Introduction

In many countries, straw is an abundant cellulosic by-product from the production of crops such as wheat, corn, soybean and rice. The natural fiber comes from stalks, leaves, and seeds, such as kenaf, sisal, flax, wheat straw and rice straw [1,2]. Compared to synthetic fiber, natural fiber has many advantages such as biodegradability, flammability and non-toxicity [3,4]. Rice straw is a potential source of energy and also is a value-added by-product [5]. It represents around 45% of the volume in rice production, producing the largest quantity of crop residue. Rice straw has the most amount of cellulose from agricultural crop residues because its composition is cellulose (38.3%), hemicelluloses (31.6%) and lignin (11.8%) [6]. It has traditionally been used as animal feed for cattle, feedstock for the paper industry or organic fertilizer by burning it on the open field or burying it on to the soil [7]. The rice straw has traditionally been removed from the field by the practice of open-field burning. This practice clears the field for new plantings and cleans the soil of disease-causing agents [8].

Recent advances in nanotechnology have made the nanoscience field a hot area of research and one of the most researched areas of science in the past two decades. In general, NPs are described as particles having diameter sizes less than or equal to 0.1 µm (100 nm) and with specific properties that depend mainly on their size [9]. NP research has become an area of attraction due their unique superior properties when compared to their bulk materials, [2] which results in their wide range of applications in different fields. Some of these properties include; catalytic properties, thermal properties, electrical conductivity, optical properties, and biological applications [10,11,12,13]. Their favorable properties are influenced by their high surface energy with a high surface area to volume ratio and relatively small sizes [14]. Syntheses of NPs in polymer media have been promising due to their ease of processing, solubility, less toxicity and also because of the possibility of controlling the growth of the resulting NPs [15].

Magnetite (Fe_3_O_4_) particles present a very interesting type of magnetic materials that has attracted intensive interest in recent years due to their potential applications in various fields, such as ferrofluids, catalysts, high-density magnetic recording media and medical diagnosis [16,17]. In recent years, the usage of magnetic nanoparticles has attracted significant interest in biomedicine and biomedical engineering for applications, including magnetic carriers for drug delivery systems and contrast enhancement agents in magnetic resonance imaging (MRI) for diagnostics. The motivation for the use of magnetic nanoparticles is related to their super paramagnetic characteristics, higher saturation magnetization, and good biocompatibility. Zhang *et al*. synthesized a novel magnetic drug-targeting carrier characterized by a core-shell structure. The carrier is composed of cross-linked dextran grafted with a poly(*N*-isopropylacrylamide-*co-N,N*-dimethylacrylamide) [dextran-*g*-poly(NIPAAm-*co*-DMAAm)] shell and super paramagnetic Fe_3_O_4_ core [18].

Yuan *et al.*, describe a magnetic nanoparticle drug carrier for controlled drug release that responds to the change in external temperature or pH [19]. In similar work Misra fabricated a novel temperature and pH-responsive magnetic nano-carrier that combines tumour targeting and controlled release [20]. In addition to controlled release, these carriers simultaneously offer the possibility of imaging the delivery process by magnetic resonance imaging.

Over the past decades, researchers have proposed several synthesis methods for preparing Fe_3_O_4_-NPs, such as microemulsions, co-precipitation of an aqueous solution of ferrous and ferric ions by a base and sol–gel method [21], sonochemistry [22], colloidal method [23], nonaqueous route [24], pyrolysis reaction, *etc* [25].

Magnetite (Fe_3_O_4_) is a common magnetic iron oxide that has a cubic inverse spinel structure with fcc close packed oxygen anions and Fe cations occupying interstitial tetrahedral and octahedral sites [26]. Due to its strong magnetic and semiconducting properties, magnetite (Fe_3_O_4_) is one of the preferred well-known filler materials, which is combined with polymers/nanocomposites to be used as magnetic recording media, and in medical applications [27]. Therefore, magnetite has the potential for providing the desired magnetic and electrical properties to the final composite. 

In this work, rice straw/Fe_3_O_4_-NCs were prepared at room temperature in aqueous media using ferric chloride, ferrous chloride, sodium hydroxide and urea as an iron oxide precursors, reducing agent, and stabilizer respectively. To our knowledge, this is the first report on the synthesis and characterization of rice straw/Fe_3_O_4_-NCs.

## 2. Results and Discussion

**Figure 1:**
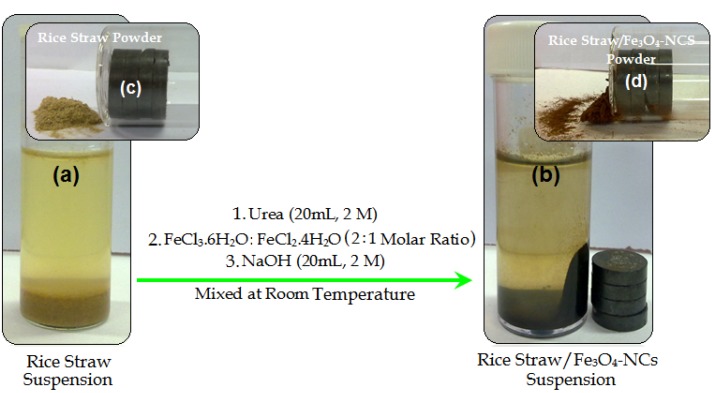
Schematic illustrate of rice straw, and rice straw/Fe_3_O_4_-NCs suspensions (**a**,**b**) and powders (**c**,**d**).

As shown in Figure 1, the rice straw suspension was brown in colour, and after the addition of the FeCl_3_·6H_2_O and FeCl_2_·4H_2_O to the rice straw suspension and addition of NaOH solution as reducing agent the suspension turned to a dark colour. Conventionally, magnetite Fe_3_O_4_-NPs are prepared by adding a base to an aqueous mixture of Fe^3+^ and Fe^2+^ chloride at a 2:1 molar ratio.

The chemical reaction of Fe_3_O_4_ precipitation is given in below Equations (1) and (2). The overall reaction may be written as follows [17,28]:

(1) $$\text{Rice straw}+{{\text{H}}_{2}\text{O}}_{\left(\text{L}\right)}+2{\text{Fe}}_{\left(\text{aq}\right)}^{3+}+{\text{Fe}}_{\left(\text{aq}\right)}^{2+}\overset{\mathrm{Stirring}}{\to }[\text{Rice straw}/2{\text{Fe}}^{3+}:1{\text{Fe}}^{2+}]$$

(2) $$[\text{Rice straw}/2{\text{Fe}}^{3+}:1{\text{Fe}}^{2+}]+{8\text{OH}}_{\left(\text{aq}\right)}^{-}\to [\text{Rice straw}/{\text{Fe}}_{3}{\text{O}}_{4}]{↓}_{\left(\text{s}\right)}+\;4{{\text{H}}_{2}\text{O}}_{\left(\text{L}\right)}$$

The comparison between the PXRD patterns of rice straw and the rice straw/Fe_3_O_4_-NCs prepared by the chemical reduction route indicated the formation of Fe_3_O_4_-NPs on the surface of the rice straw. TEM images were used to understand the morphology of the nanocomposites. The TEM images of rice straw/Fe_3_O_4_-NCS showed that the mean diameter and standard deviation of the NPs were about 9.93 ± 2.42 nm. The FESEM images showed good dispersion of Fe_3_O_4_-NPs on the surface of rice straw. Additionally, the FESEM images indicated that there were no structural changes between the rice straw and rice straw/Fe_3_O_4_-NCs. The FT-IR results of prepared NCs showed that the Fe_3_O_4_-NPs were successfully coated on the surface of rice straw.

### 2.1. Powder X-ray Diffraction

**Figure 2:**
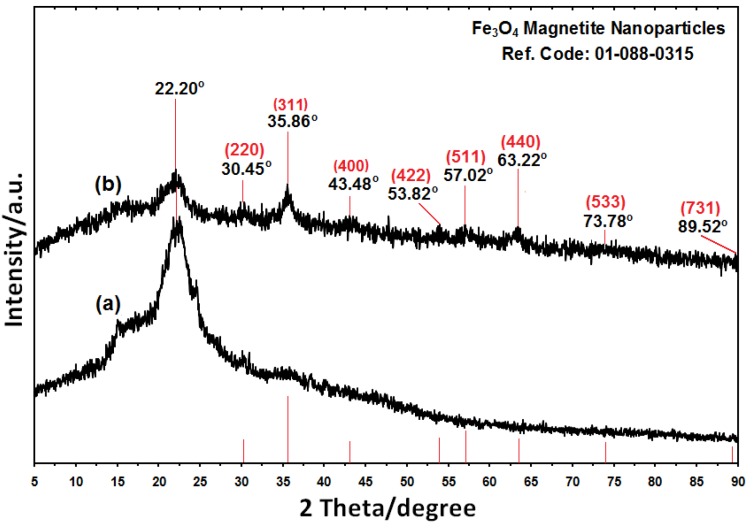
The PXRD of rice straw and rice straw/Fe_3_O_4_-NCs with the related peaks respectively (a,b).

The comparison between the PXRD patterns of the rice straw and rice straw/Fe_3_O_4_-NCs in the small angle range of 2θ (15-25) indicated the formation of the intercalated Fe_3_O_4_ nanostructure Figure 2a,b. In addition to the broad diffraction peak, which was centered at 22.20° is attributed to rice straw, eight crystalline peaks were observed at 2θ° of 30.45°, 35.86°, 43.48°, 53.82°, 57.02°, 63.22°, 73.78° and 89.52° related to the 220, 311, 400, 422, 511, 440, 533 and 731 crystallographic planes of face-centered cubic (fcc) iron oxide nanocrystals, respectively (Ref. Code Fe_3_O_4_: 01-088-0315) [29,30,31].

The effect of magnetic field on the rice straw and rice straw/Fe_3_O_4_-NCs powder is shown in Figure 1c,d. These results confirmed that there are significant amount of Fe_3_O_4_-NPs on the surface of rice straw because the Fe_3_O_4_-NPs prepared in rice straw/Fe_3_O_4_-NCs attracted by magnetic [32]. The presence of Fe_3_O_4_on the surface of rice straw fiber gives wider peaks with low intensity which show increase in the amorphous characteristics of the NCs with iron contents [33]. The average particle size of Fe_3_O_4_-NPs in rice straw can be calculated using Scherrer’s Equation (3):

n = K*λ*/β cos θ (3)

where K is the Scherrer’s constant with value from 0.9 to 1 (shape factor), where λ the X-ray wavelength (1.5418 Å), β1/2 is the width of the XRD peak at half height and θ is the Bragg angle. From the Scherrer’s equation, the average crystallite size of Fe_3_O_4_-NPs for rice straw is found to be less than 10 nm, which was also in line with the observation of the TEM and FESEM results discussed later. 

### 2.2. Transmission Electron Microscopy

**Figure 3:**
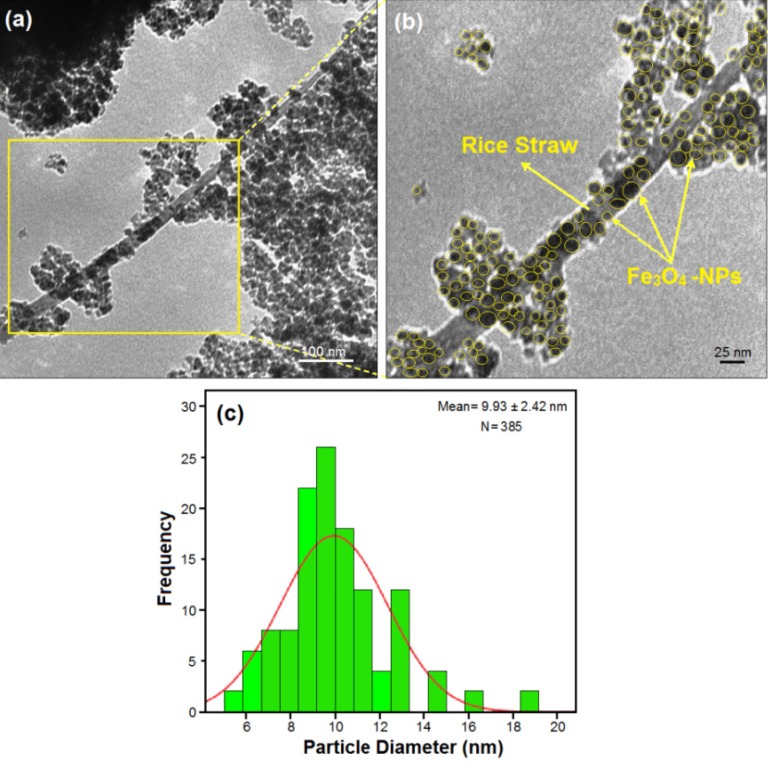
Transmission electron microscopy images and histogram (**a**,**b**) of particle size distribution for rice straw/Fe_3_O_4_-NCs (**c**).

TEM images and size distribution of rice straw/Fe_3_O_4_-NCs containing of Fe_3_O_4_-NPs and calculated histogram are shown in Figure 3. The TEM images and their size distributions showed that the mean diameters and standard deviation of Fe_3_O_4_-NPs were about 9.93 ± 2.42 nm. 

In addition, this confirms the uniform distribution of the Fe_3_O_4_-NPs in the rice straw matrix, although particles seem to aggregate to some extent. It can be seen that the Fe_3_O_4_-NPs exhibit spherical morphology, which agreed well with the results of XRD. Importantly, no morphological differences were observed between the rice straw and rice straw/Fe_3_O_4_-NCs. The numbers of Fe_3_O_4_-NPs counted for TEM image were around 385.

### 2.3. Scanning Electron Microscopy

**Figure 4:**
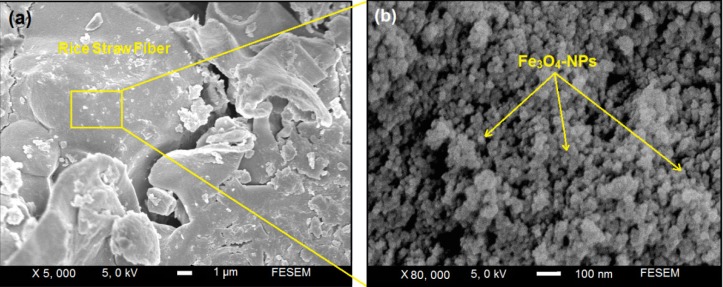
Scanning electron microscopy micrographs of rice straw/Fe_3_O_4_-NPs with low and high magnification (**a**,**b**).

The Fe_3_O_4_-NPs prepared on the surface of rice straw are shown by SEM in Figure 4 and confirmed that the structure of rice straw have not particular change. Figure 4a,b show the SEM images for the rice straw/Fe_3_O_4_-NCs synthesized by the quick precipitation method. These results confirm that the modify surface of rice straw can effectively control shape and size of the Fe_3_O_4_-NPs. The surfaces of rice straw/Fe_3_O_4_-NCs with high magnification gradually become shiny, due to the presence of small size of Fe_3_O_4_-NPs those aggregation together and created large particles (Figure 4b).

### 2.4. Energy Dispersive X-ray Spectroscopy

**Figure 5:**
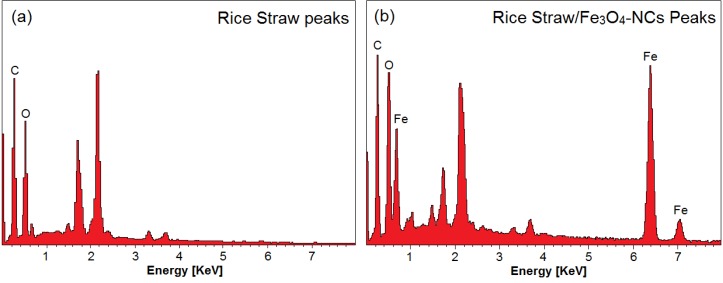
Energy dispersive X-ray spectroscopy of rice straw and rice straw/Fe_3_O_4_-NCs Peaks (**a**,**b**).

Energy dispersive X-rays (EDX) was used to analyze the elemental constituents of the rice straw and rice straw/Fe_3_O_4_-NCs (Figure 5).

Figure 5a shows the EDXRF spectra for the rice straw, the peaks around 0.23, 0.50, 0.62, 1.21, 1.44, 1.71, 2.18, 2.20, 3.30 and 3.70 keV are related to the binding energies of rice straw. In Figure 5b 0.68, 6.20 and 7.30 keV related to Fe_3_O_4_-NPs elements, respectively [34]. Additionally, the EDXRF spectra for the rice straw and rice straw/Fe_3_O_4_-NCs confirm the presence of elemental compounds in the rice straw and Fe_3_O_4_-NPs without any impurity peaks. The results indicate that the synthesized Fe_3_O_4_-NPs are of high purity.

### 2.5. FT-IR Chemical Analysis

**Figure 6:**
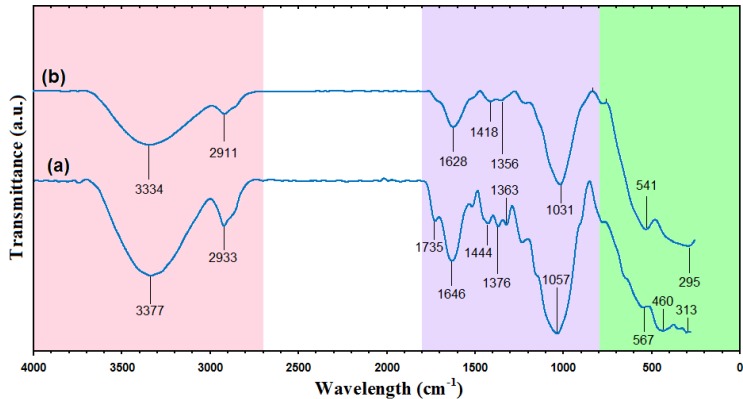
Fourier transforms infrared spectra of rice straw and rice straw/Fe_3_O_4_-NCs (a,b).

Figure 6 shows the FT-IR spectra of rice straw and rice straw/Fe_3_O_4_-NCs with NaOH. In the FT-IR spectrum of raw rice straw Figure 6a, the absorption peaks at 3,377 cm^−1^ and 2,933 cm^−1^ are ascribed to stretching vibrations of –OH groups and the C‒H stretching, respectively [7]. The smaller shoulder peak at 1,735 cm^−1^ in the rice straw is attributed to the aliphatic esters in lignin or hemicelluloses. The intense band at 1,646 cm^−1^ is assigned to olefinic C=C stretching vibration [35]. The peak at 1,444 cm^−1^ is ascribed to the aromatic C=C stretch of aromatic vibration in bound lignin [7]. The absorbance peaks in the 1,363–1,376 cm^−1^ originate from C-H bending [36]. The region of 1,000–1,200 cm^−^^1^ represents C–O stretch and deformation bands in cellulose, lignin and residual hemicelluloses [37]. The small peaks in 313‒567 cm^−1^ represent Si-O-Si stretching in silica [38].

The presence of Fe_3_O_4_-NPs on the surface of rice straw evidenced by the adsorption bands at around 295‒541cm^−1^ that confirm the Fe‒O stretching are shown in Figure 5b [26]. Two bands at 1,628 and 1,418 cm^−1^ showed a reaction between hydroxyl groups on the surface of Fe_3_O_4_-NPs and carboxylate groups of urea [39]. Absorption peak at 3,334 cm^−1^, the associated hydroxyl groups, indicated the existence of rice straw [40].

In comparison with rice straw, rice straw/Fe_3_O_4_-NCs show decrease in the intensity of the adsorption peaks, the possible reason for this decrease in intensity is that rice straw has been partially reduced [34]. This result indicated that Fe_3_O_4_-NPs be successfully coated on the surface of rice straw.

## 3. Experimental

### 3.1. Materials and Methods

All chemical agents used in this study were of analytical grade and used without further purification. Rice straw was obtained from local farm (Bukit Tinggi, Kedah, Malaysia). Materials used for the synthesis of Fe_3_O_4_-NPs included FeCl_3_·6H_2_O and FeCl_2_·4H_2_O (99.89%) were supplied by Merck (Frankfurt, Germany), Urea (99%) was purchased from Hamburg Chemicals (Hamburg, Germany), NaOH (99.0%), obtained from R & M Chemistry (Chicago, IL, USA), while HNO_3_ (70%) and HCl (37%) were obtained from Sigma-Aldrich (St. Louis, MO, USA). All solutions were freshly prepared using double distilled water and kept in the dark to avoid any photochemical reactions. All glassware used in experimental procedures were cleaned in a fresh solution of HNO_3_/HCl (3:1, v/v), washed thoroughly with double distilled water, and dried before use.

### 3.2. Synthesis of Rice Straw/Fe_3_O_4_ Nanocomposites

For the synthesis of rice straw/Fe_3_O_4_-NCs, rice straw (2.0 g) was suspended in double distilled water (60 mL), then prepared urea solution (20 mL, 2.0 M) was added into the mixture as stabilizer. The FeCl_3_·6H_2_O and FeCl_2_·4H_2_O (2:1 molar ratio) were added into the modified rice straw suspension with vigorous stirring under nitrogen gas to prevent oxidation. Then freshly prepared NaOH (20 mL, 2.0 M) was added into the mixture under continuous stirring till a black suspension was formed Figure 1a,b. The suspension was finally centrifuged, washed twice with ethanol and deionized water and dried in oven at 60 °C. All the experiments were conducted at ambient temperature. Moreover as shown in the Figure 1c the magnet does not attract pure rice straw powder, while when the Fe_3_O_4_-NPs was formed on the surface of rice straw, the resulting rice straw/Fe_3_O_4_-NCs are attracted by the magnet Figure 1d.

### 3.3. Characterization Methods and Instruments

Transmission electron microscopy (TEM) was used to measure the morphology and size of samples obtained. A drop of diluted sample in distilled water was dripped onto a covered copper grid. The TEM observations were carried out using a Hitachi H‒7100 electron microscope, and the particle size distributions were determined using the UTHSCSA Image Tool software, version 3.00 program. Electron field emission scanning electron microscopy (FESEM) was applied to observe morphology of rice straw and rice straw/Fe_3_O_4_-NCs. The FESEM with Energy dispersive X-ray fluorescence (EDXF) spectroscopy was performed utilizing a JEOL, JSM‒7600F instrument. The powder X-ray diffraction (PXRD) with Cu K_α_ radiation was used to measure the crystallinity of samples. Fourier transform infrared (FT-IR) in the range of 400–4,000 cm^−1^ was used in order to study the structures of the rice straw, urea and rice straw/Fe_3_O_4_-NCs. FT-IR spectra were recorded utilizing a Series 100 Perkin Elmer FT-IR 1650 spectrophotometer.

## 4. Conclusions

Novel rice straw/Fe_3_O_4_-NCs were prepared by quick precipitation of ferrous and ferric ions using sodium hydroxide as a precipitating agent. The reduction of Fe^3+^and Fe^2+^ ions by NaOH as a reducing agent followed by the flow of N_2_ gas at ambient temperature and under a non-oxidizing oxygen-free environment. Rice straw/Fe_3_O_4_-NCs with a mean diameter and standard deviation of 9.93 ± 2.42 nm were prepared for the first time, whichs agrees well with particle size determined from XRD patterns. The SEM images showed good dispersion of Fe_3_O_4_-NPs in rice straw. The FT-IR results of prepared nanocomposites showed that the Fe_3_O_4_-NPs were successfully coated on the surface of rice straw. This method is a cheap and environmentally friendly one that leads to preparation of rice straw/Fe_3_O_4_-NCs; the Fe_3_O_4_-NPs prepared by this method were also attracted by magnets. This indicates that the metal oxides NPs were coated on the surface of rice straw.
